# Medical Students’ Perception of Interprofessional Education: A Cross-Sectional Study

**DOI:** 10.7759/cureus.50501

**Published:** 2023-12-14

**Authors:** Khaled J Alghamdi, Reema M Aljohani, Rahaf A Khurmi, Jumana A Alrehaili, Yara M Alrehaili, Roza E Allam, Asmaa R Aljohani

**Affiliations:** 1 Department of Pediatrics, Taibah University, Madinah, SAU; 2 Faculty of Medicine, Taibah University, Madinah, SAU

**Keywords:** teamwork, education, medical, learning, interprofessional

## Abstract

Background and objective

Interprofessional education (IPE) involves learners from multiple health professions learning collaboratively to improve patient care. This study assessed medical students' perceptions of IPE at Taibah University in Saudi Arabia.

Methods

In this cross-sectional study, 319 medical students in years two to six of graduate medical school and internships completed the Readiness for Interprofessional Learning Scale (RIPLS) questionnaire between April 2022 and July 2022. RIPLS consists of 19 items measuring teamwork/collaboration, negative/positive professional identity, and roles/responsibilities. Comparisons were made based on gender and academic level by using the Mann-Whitney U and Kruskal-Wallis tests.

Results

Students generally expressed positive attitudes towards IPE. The majority agreed that IPE improves teamwork, communication, and patient care. In our cohort, 148 students (46.4%) strongly agreed and 140 (43.9%) agreed that shared learning enhances understanding of clinical problems. However, 186 students (60%) disagreed that clinical problem-solving is profession-specific. On the teamwork/collaboration subscale, 279 students (87.7%) strongly agreed that learning with others makes them more effective team members. Regarding negative identity, 186 students (58.3%) disagreed that learning with other students wastes time. By gender, males had lower ranks for negative identity (p=0.03) and positive identity (p=0.03) versus females. As for academic level, clinical students and interns had higher and lower ranks, respectively, for negative identity (p<0.01).

Conclusion

Based on our findings, medical students generally hold favorable views toward IPE and recognize its benefits for collaboration, communication, and patient care. Incorporating IPE throughout medical training may further improve attitudes and interprofessional skills.

## Introduction

An interprofessional team comprises members from different health professions with different specialized knowledge, skills, and abilities. Interprofessional education (IPE) involves educators and learners from multiple health professions or disciplines. These participants collectively construct an interactive learning space that fosters the growth of knowledge, abilities, and attitudes, which in turn culminate in interprofessional team behaviors and proficiencies. In an ideal setting, IPE is seamlessly woven both vertically and horizontally into the entire educational program [1,2]. The objective of IPE among undergraduate students is to instruct them on functioning effectively within an interprofessional team. They learn to effectively employ their acquired knowledge, abilities, and values in providing patient care collaboratively to enhance patient outcomes [3].

IPE constitutes an essential step in advancing healthcare professional education and has been evaluated from different perspectives in different countries in several professions with a view to improving the quality of healthcare. In a review, nearly half of the reports showed a significant increase in positive attitudes toward interprofessional cooperation [4]. Nevertheless, varying attitudes towards IPE were noted among different academic levels. In a study from Thailand, a huge collaborative effort involving medical, pharmacy, architecture, urban design, and creative arts students demonstrated that their perceptions of collaboration and teamwork significantly improved after engaging in IPE practices; these improvements were positively reflected in their performance in IPE home-based care [5].

An IPE model in India in the fields of medicine, nursing, and physiotherapy greatly improved participants’ levels of comfort and dependence on other specialists [6]. Patient safety-directed IPE regarding prehospital care, infection control, and medication errors was provided to different cohorts and led to satisfactory outcomes [7,8,9]. In the era of the information revolution and major advances in social media, extraordinary collaborations among medical schools and other professions, such as engineering, facilitate the development of unique healthcare solutions that are in high demand. Such collaboration in medical, nursing, and public health fields can improve health advocacy and veterinary medicine to facilitate contributions to stem cell research and water and food safety [10,11,12]. IPE has been shown to be beneficial in many contexts globally. However, perceptions of and readiness for IPE likely vary across cultural and institutional contexts. There have been scarce studies on IPE in Saudi Arabia. In light of this, this study aimed to assess the perceptions of and readiness for the interprofessional learning process among medical students at different educational levels at Taibah University. Assessing readiness for IPE is an essential step in advancing healthcare professional education in this institutional and cultural context. We believe our findings will provide valuable insights into the benefits and challenges of implementing IPE curricula at Taibah University and in Saudi Arabia more broadly.

## Materials and methods

Study design, setting, and participants

This cross-sectional study aimed to assess the perception of IPE among medical students at Taibah University, Madinah, Saudi Arabia. The study was conducted from April 2022 to July 2022; medical students were classified into three categories: students in basic years (second and third years), which involve foundational courses not taught solely by the faculty of medicine; students in clinical years (fourth to sixth years); and students in the internship year (following the sixth year). The online link to the questionnaire was shared among medical students in the second, third, fourth, fifth, sixth, and internship years at the Faculty of Medicine, Taibah University, Madinah, Saudi Arabia.

Variables

We assessed medical students by using the Readiness for Interprofessional Learning Scale (RIPLS), a validated, publicly available, and widely used tool to assess IPE [13]. The RIPLS is composed of 19 components, categorized into four subsets: (1) teamwork and collaboration, which includes items 1-9 with a maximum score of 45; (2) negative professional identity (NPI), encompassing items 10-12 with a maximum score of 15; (3) positive professional identity (PPI), covering items 13-16 with a maximum score of 20; and (4) roles and responsibilities, involving items 17-19 with a maximum score of 15. Each item is answered on a 5-point Likert scale, and the responses range from “strongly disagree (1)” to “strongly agree (5).” Reliability analysis was conducted on the four RIPLS subscales identified and the total score. The values for Cronbach’s alpha for the questionnaire subscales were 0.82 for teamwork and collaboration, 0.79 for NPI, 0.77 for PPI, and 0.38 for roles and responsibilities. The reliability of the total RIPLS score was 0.72.

Statistical analysis

We used SPSS Statistics version 26.0 (IBM Corp., Armonk, NY) for data analysis. Continuous data were presented as the mean and standard deviation (SD), and categorical data were presented as numbers and percentages. The normality of data was tested using the Kolmogorov-Smirnov test. Comparisons of students’ mean RIPLS subscale scores based on demographic variables were conducted using the Mann-Whitney U test (for two groups) and the Kruskal-Wallis H test (for more than two groups). Cronbach’s alpha coefficients were computed to evaluate the internal consistency of RIPLS. A p-value of less than 0.05 was considered statistically significant.

Ethical considerations

The study was approved by the Research Ethical Committee of Taibah University (study ID: STU-21-017). Participation was voluntary. All participating students provided electronic consent before they accessed the questionnaire. All students were allowed to respond privately at their own pace.

## Results

The study population consisted of 319 individuals. Of these, 96 (30.1%) were male and 223 (69.9%) were female. When categorized by academic level, 54 (16.9%) were second-year students, 57 (17.9%) were third-year students, 68 (21.3%) were fourth-year students, 54 (16.9%) were fifth-year students, 51 (16.0%) were sixth-year students, and 35 (11.0%) were interns. The study population was predominantly female and included students at various stages of their academic programs and interns. The largest subgroups were fourth-year students at 21.3% and females at 69.9% of the total study population (Table 1).

**Table 1:** Demographic characteristics of the study population (N=319)

Characteristic	Frequency	Percentage
Gender		
Male	96	30.1%
Female	223	69.9%
Academic level		
Second year	54	16.9%
Third year	57	17.9%
Fourth year	68	21.3%
Fifth year	54	16.9%
Sixth year	51	16.0%
Intern	35	11.0%

Table 2 illustrates medical students' attitudes toward IPE across three domains: teamwork and collaboration, and negative and positive professional identities. Students overwhelmingly agreed that learning with other healthcare students promotes teamwork and communication and improves patient care. In the cohort, 148 students strongly agreed (46.4%) and 140 students (43.9%) agreed that shared learning would enhance their ability to understand clinical problems. However, some students held negative views about IPE. Nearly 60% (n=186) disagreed that clinical problem-solving skills can only be learned with students from their departments. In the positive identity domain, most students (over 80%, n=257) believed shared learning would help them communicate better with patients and become more effective team members. There were some uncertainties regarding professional roles and responsibilities. While 196 students (61.8%) agreed that they needed to acquire more knowledge and skills than other healthcare students, 115 (36.1%) disagreed that they were unsure of their professional role. Our results indicate that students have predominantly positive attitudes toward IPE and acknowledge its benefits in terms of collaboration, communication, and patient care.

**Table 2:** Medical students’ responses to the RIPLS questionnaire RIPLS: Readiness for Interprofessional Learning Scale

	Strongly agree		Agree		Neutral		Disagree		Strongly disagree		Mean	Mean category
	N	%	N	%	N	%	N	%	N	%		
Teamwork and collaboration												
1. Studying alongside other students will enhance my abilities to become a more productive healthcare team member	132	41.4%	138	43.3%	39	12.2%	9	2.8%	1	0.3%	4.2	Strongly agree
2. If healthcare students collaborated to address patient issues, it would eventually lead to improved outcomes for the patients	147	46.1%	132	41.4%	36	11.3%	3	0.9%	1	0.3%	4.3	Strongly agree
3. Collaborative learning with other healthcare students will enhance my capacity to comprehend clinical issues	148	46.4%	140	43.9%	27	8.5%	2	0.6%	2	0.6%	4.3	Strongly agree
4. Engaging in educational experiences alongside students in healthcare fields before completing their studies enhances interpersonal dynamics post-graduation	136	42.6%	146	45.8%	32	10.0%	5	1.6%	0	0%	4.3	Strongly agree
5. Developing communication abilities in conjunction with peers from various healthcare disciplines is essential	135	42.3%	136	42.6%	43	13.5%	5	1.6%	0	0%	4.3	Strongly agree
6. Collaborative education fosters positive perceptions toward fellow professionals	121	37.9%	143	44.8%	47	14.7%	6	1.9%	2	0.6%	4.2	Agree
7. Effective small-group learning hinges on mutual trust and respect among students	206	64.6%	89	27.9%	18	5.6%	5	1.6%	1	0.3%	4.5	Strongly agree
8. It is vital for healthcare students to acquire teamwork competencies	168	52.7%	113	35.4%	32	10.0%	6	1.9%	0	0%	4.4	Strongly agree
9. Engaging in collaborative education aids in recognizing personal limitations	110	34.5%	158	49.5%	40	12.5%	10	3.1%	1	0.3%	4.1	Agree
Negative professional identity												
10. I prefer not to spend time in joint educational sessions with students from other healthcare fields	13	4.1%	45	14.1%	75	23.5%	140	43.9%	46	14.4%	2.5	Disagree
11. Joint learning for undergraduate students in healthcare is not imperative	12	3.8%	55	17.2%	75	23.5%	125	39.2%	52	16.3%	2.5	Disagree
12. Developing clinical problem-solving abilities is exclusive to learning within my department	27	8.5%	53	16.6%	91	28.5%	117	36.7%	31	9.7%	2.8	Neutral
Positive professional identity												
13. Collaborative education with peers from various healthcare disciplines enhances my communication skills with patients and professionals	107	33.5%	160	50.2%	42	13.2%	9	2.8%	1	0.3%	4.1	Agree
14. I am open to engaging in small group projects with students from different healthcare fields	91	28.5%	167	52.4%	47	14.7%	13	4.1%	1	0.3%	4.0	Agree
15. Collaborative education aids in better understanding patient issues	95	29.8%	172	53.9%	45	14.1%	4	1.3%	3	0.9%	4.1	Agree
16. Engaging in collective learning before graduation will enhance my teamwork skills	125	39.2%	154	48.3%	36	11.3%	3	0.9%	1	0.3%	4.3	Strongly agree
Roles and responsibilities												
17. Nurses and therapists primarily serve in roles that assist doctors	44	13.8%	96	30.1%	89	27.9%	63	19.7%	27	8.5%	3.2	Neutral
18. I am uncertain about my future professional responsibilities	25	7.8%	71	22.3%	86	27.0%	115	36.1%	22	6.9%	2.9	Neutral
19. My education requires me to gain broader knowledge and skills compared to other healthcare students	59	18.5%	138	43.3%	81	25.4%	37	11.6%	4	1.3%	3.7	Agree

Table 3 compares RIPLS subscale scores based on students' demographics using mean ranks and p-values. Regarding gender, males had a significantly lower mean rank than females for NPI (174.9 vs. 150.6, p=0.03). Females also had a higher mean rank for PPI (165.1 vs. 141.5, p=0.03). As for academic level, clinical students had a higher mean rank than basic students for negative identity (167.5 vs. 144.1, p<0.01). Interns had the lowest mean rank on this subscale (118.9). Concerning roles and responsibilities, there were significant differences between academic levels (p<0.01), with interns having the lowest mean rank (105.8). Comparing sixth-year students and interns, interns had a significantly lower mean rank for NPI (29.5 vs. 48.8, p<0.01). Overall, the results show differences in attitudes based on gender and academic level, with females and more senior clinical students reporting more positive perspectives.

**Table 3:** Comparison of RIPLS subscales based on students’ demographics *Mann-Whitney U test. **Kruskal–Wallis test: p-value <0.05 is statistically significant RIPLS: Readiness for Interprofessional Learning Scale

Variable	Category	Teamwork and collaboration		Negative professional identity		Positive professional identity		Roles and responsibilities	
		Mean rank	P-value	Mean rank	P-value	Mean rank	P-value	Mean rank	P-value
Gender	Male	144.3	0.05	174.9	0.03*	141.5	0.03*	172.4	0.11
	Female	166.1		150.6		165.1		154.7	
Academic level	Basic	158.4	0.72	144.1	<0.01**	162.3	0.67	160.4	<0.01**
	Clinical	156.9		167.5		155.1		161.8	
	Intern	170.4		118.9		167.8		105.8	
Before and after the internship	Sixth year	40.5	0.37	48.8	<0.01*	40.9	0.23	47.3	0.05
	Intern	45.3		29.5		47.3		36.9	

## Discussion

Our findings showed overall strong agreement among medical students at different academic levels on the importance of IPE. Participants broadly agreed about the value of and need for collaborative education. Medical students in their clinical years expressed more positive attitudes toward IPE overall compared to students in basic years; however, there was no significant difference between the two groups concerning their understanding of professional roles and responsibilities specifically. This aligns with the findings of Mark et al. who found that students with less experience in the healthcare system scored worse on the NPI subscale [14,15]. This outcome may be attributed to the fact that most participants had no direct interaction with their coworkers or the healthcare system. Similarly, we found a statistically significant difference between primary, clinical, and internship students regarding IPE.

Additionally, we found that students, especially male students in clinical years, had negative professional attitudes, which included a lack of awareness about the benefits of teamwork and the ability to solve problems. Finally, female students agreed on the importance of IPE in enhancing teamwork and communication skills. These findings were consistent with those of Alzamil and Meo [16].

In this study, male students' negative views of IPE were influenced by psychological and social factors. Males' learning styles lean towards individualism due to gender differences in socialization. They prefer competitive learning environments, while females lean towards cooperation. Medical field stereotypes, which see males as independent and females as team supporters, reinforce these behaviors. In Saudi Arabia, cultural norms discourage males from expressing uncertainties, which might undermine their competence if they admit knowledge gaps or validate others' roles. The male-dominated medical profession promotes self-reliance, fostering negative attitudes toward sharing expertise or ceding control to other disciplines. Due to their privileged position, male students may resist the collaborative nature of IPE. Their lack of prior experience in such collaborative efforts could make them feel threatened by a perceived loss of authority [15,16].

Our sample size was much larger compared to similar studies conducted in Saudi Arabia. Moreover, most participants valued IPE as a tool for enhancing their interprofessional skills. The findings of a previous study at Taibah University were consistent with ours [15]. It involved 40 students from the beginning (first month) to the end (final month) of the internship period. The interns were from the applied medical sciences college in the diagnostic radiologic technology, medical laboratory sciences, and clinical nutrition departments. In the study, most of the internship students (88.7%) agreed that IPE could enhance their collaborative and teamwork skills. A similar study was conducted among 158 medical students at King Saud University. Most of the participants agreed that IPE positively impacted teamwork and collaboration (122-148, 77-94%). Over two-thirds of participants (105, 64.45%) disagreed with negative attitude statements, and 70%-80% showed PPI. Most participants agreed that sharing learning with other healthcare professionals would help them communicate better with patients and other professionals and improve their practice [15-19]. Various researchers have attributed the lack of collaboration among health professionals to misunderstandings of their colleagues' various roles and scopes of practice [17,18].

In Saudi Arabia, students in various healthcare professions have a positive attitude and are ready and eager for collaborative learning [20]. They are also well prepared for IPE and willing and keen to polish their leadership and communication skills and explain their professional duties and limitations [21]. A prospective, controlled study found that a brief IPE intervention program significantly improved students’ attitudes toward interprofessional learning and enhanced self-reported confidence and efficacy as a part of the healthcare team [22].

Our study has a few limitations. We did not evaluate the outcomes of IPE, as no follow-up was conducted due to the cross-sectional design of the study. Moreover, we had an unequal distribution of samples, particularly between genders.

## Conclusions

This study assessed medical students' perceptions of IPE at Taibah University in Saudi Arabia. Our findings showed that students held positive attitudes toward IPE. Most of them agreed that IPE improves teamwork, communication skills, and patient care. However, some uncertainties existed regarding professional roles and responsibilities. Comparisons based on demographics found that females and more senior clinical students tended to have more favorable perspectives on IPE versus males and junior students. Our findings indicate that medical students acknowledge the benefits of IPE in enhancing collaboration, communication, and care delivery. Incorporating IPE throughout medical education could improve students' interprofessional skills and attitudes. Further research is needed to evaluate the impact of IPE interventions on tangible outcomes.
